# Non-thermal plasma-induced apoptosis is modulated by ATR- and PARP1-mediated DNA damage responses and circadian clock

**DOI:** 10.18632/oncotarget.9087

**Published:** 2016-04-28

**Authors:** Ji Ye Choi, Hea Min Joh, Jeong-Min Park, Min Ji Kim, Tae Hun Chung, Tae-Hong Kang

**Affiliations:** ^1^ Department of Biological Science, Dong-A University, Busan 604714, Republic of Korea; ^2^ Department of Physics, Dong-A University, Busan 604714, Republic of Korea

**Keywords:** non-thermal plasma, DNA damage response, ATR, PARP1, circadian clock

## Abstract

Non-thermal plasma (NTP) has been emerging as a potential cancer therapeutic. However, the practical use of NTP as a cancer therapy requires a better understanding of the precise mechanisms underlying NTP-induced DNA damage responses in order to achieve optimal efficacy. It has been shown that the addition of oxygen gas flow during NTP treatment (NTPO), when compared to NTP exposure alone, can induce a 2–3 fold greater generation of intracellular reactive oxygen species (ROS) in A549 cells. Here, we examined NTPO-induced DNA damage responses and found that NTPO generated a substantial number of genomic DNA lesions and breaks that activated ATR-mediated cell-cycle checkpoints. In addition, we discovered that NTPO-induced DNA lesions were primarily removed by base excision repair (BER) rather than by nucleotide excision repair (NER). Therefore, the inhibition of the BER pathway using a PARP1 inhibitor drastically induced the phosphorylation of γH2AX, and was followed by the programmed cell death of cancer cells. However, the knock-down of XPA, which inhibited the NER pathway, had no effect on NTPO-induced phosphorylation of γH2AX. Finally, in agreement with a recent report, we found a circadian rhythm of PARP1 activity in normal mouse embryonic fibroblasts that needed for cell viability upon NTPO treatment. Taken together, our findings provided an advanced NTP regimen for cancer treatment by combining NTPO treatment with chemical adjuvants for the inhibition of ATR- and PARP1-activated DNA damage responses, and circadian timing of treatment.

## INTRODUCTION

Recently, the potential usefulness of non-thermal plasma (NTP) formed at atmospheric pressure and near room temperature for biomedical applications including wound healing and sterilization, antisepsis, and cancer treatment has been shown [1–3]. It is believed that such effects are mainly driven by the reactive oxygen species (ROS) and reactive nitrogen species (RNS) generated from the exposed culture medium, as indicated by the observation that the exposed medium itself has similar effects on cellular physiologies that direct NTP exposure in cells [4]. For examples 1.6 kV of NTP treatment produced approximately 100 μM nitrite in human lung carcinoma A549 cells [5]. Excessive amounts of ROS are potentially deleterious to cells because of the interactions with cellular macromolecules including DNA [6, 7]. Furthermore, adding a small amount of oxygen gas in NTP was found to be useful in production of the ROS and RNS, and shown to inhibit tumor cell migration and invasion [8]. Indeed, we recently showed that NTP combined with oxygen gas (NTPO) generated more oxyradicals compared to NTP itself, which was followed by the activation of the proapoptotic p53 pathway in human lung cancer cells [9], which implied that NTP/NTPO might evoke specific DNA damage responses. Although NTP itself has been shown to generate DNA damage in cancer cells, the efficiency needs to be enhanced to achieve optimal therapeutic efficacy. Because apoptotic signaling is mainly activated in response to genotoxic stresses, a comprehensive understanding of NTP-induced DNA damage responses (DDR) poses the potential for utilizing NTP for therapeutic benefit.

DNA damage-induced cell-cycle checkpoints serve to monitor genomic integrity and to coordinate multiple cellular pathways to ensure efficient repair following DNA damage [10]. The ataxia telangiectasia mutated (ATM), ATR (ATM and Rad3-related), and DNA-PK-mediated checkpoint pathways represent major DNA damage checkpoints in mammals [11]. Although ATM- and DNA-PK-induced checkpoint kinase 2 (CHK2) activation is believed to orchestrate double-strand DNA break (DSB) repair, ATR-dependent CHK1 activity is necessary for sensing single-strand damage such as base oxidation [12]. The most prevalent oxidative damage to purine bases is 8-oxoguanine (8-OxoG), whereas the most common oxidative damage to pyrimidine bases is thymine glycol [13]. Such lesions can be detected and essentially restored by two types of repair systems. Base excision repair (BER) is considered the primary mechanism for removing oxidized bases, which requires the action of poly-ADP-ribose polymerase-1 (PARP1), a highly conserved nuclear protein that adds ADP-ribose molecule chains to proteins in a process called poly-ADP-ribosylation (PAR) [14, 15]. Increasing evidence indicates that nucleotide excision repair (NER) is also involved in neutralizing some types of oxidized bases [13]. NER has a wide substrate spectrum that ranges from lesions such as thymine glycols, which do not induce helix distortion, to the benzo [*a*]pyrene-guanine adducts that induce helix distortion in DNA structures [16].

In order to obtain insight into exploiting NTP for cancer therapy, we assessed NTP and NTPO-induced DNA damage responses, including cell-cycle checkpoints, DNA repair, and apoptosis in A549 and SK-MEL2 human cancer cells, which represent lung carcinoma and melanoma, respectively. Our results demonstrated that NTP and NTPO generated substantial genomic DNA breaks that subsequently activated ATR-mediated cell-cycle checkpoints and PARP1-dependent DNA repair.

## RESULTS

**Figure 1:**
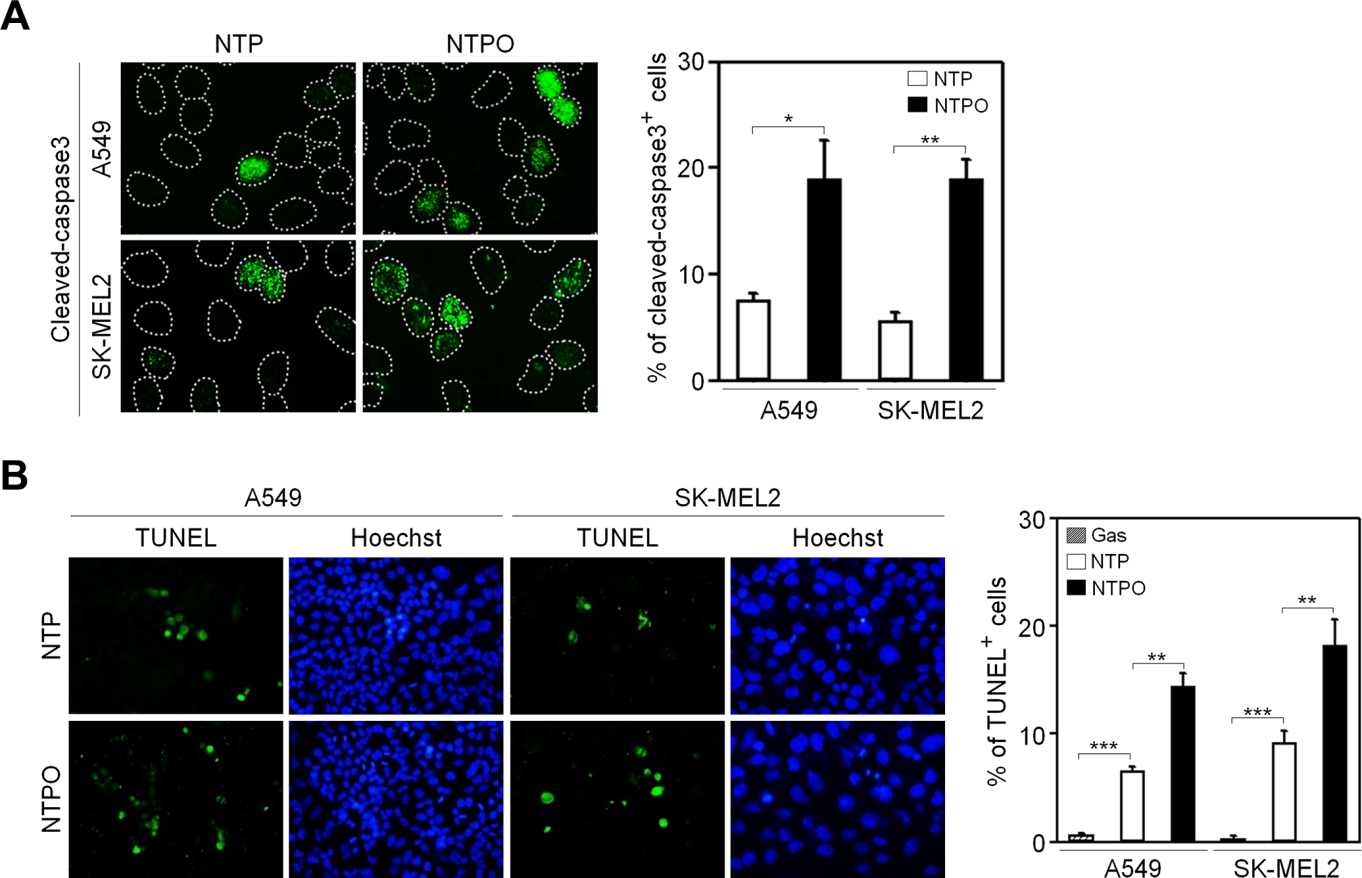
Enhanced apoptosis by the addition of oxygen gas flow during NTP treatment in human cancer cells (**A**) A549 and SK-MEL2 cells treated with NTP supplemented with or without the flow of oxygen gas were fixed and immunostained for cleaved-caspase 3, and Hoechst-stained nuclei were depicted as dotted lines. (**B**) A TUNEL assay was performed to detect DNA fragmentation. Bars and error bars represent mean ± SD from three independent experiments (* = *p* < 0.05; ** = *p* < 0.01; *** = *p* < 0.001).

**Figure 2:**
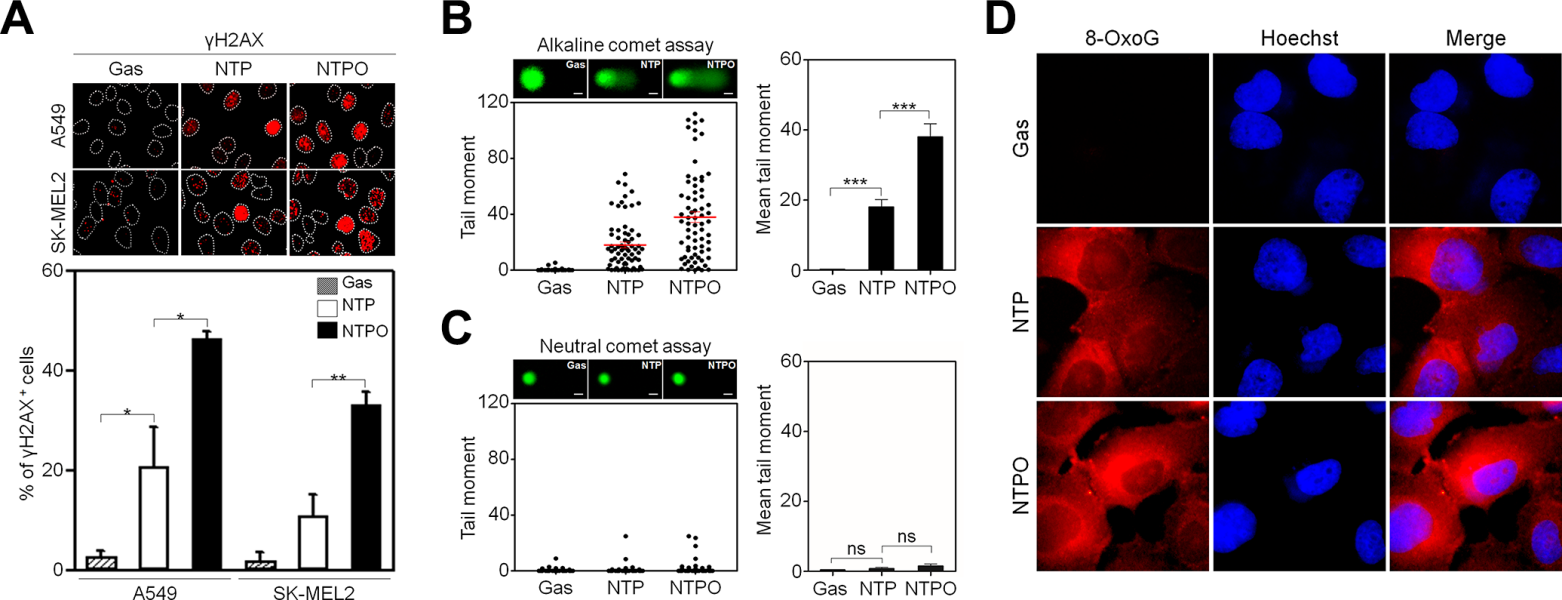
NTP and NTPO induce genomic DNA lesions and breaks (**A**) A549 and SK-MEL2 cells treated with gas control, NTP, or NTPO were fixed and immunostained for γH2AX and Hoechst-stained nuclei were depicted as dotted lines. (**B, C**) The extents of DNA breaks were assessed using the comet assay either under alkaline condition for detection of both DNA single strand and double strand breaks (B) or under neutral condition for detection of DNA double strand breaks (C). Representative comet images after 24 hours following the gas control, NTP, and NTPO treatment were presented. The tail moment obtained from the comet assay was analyzed quantitatively. Scale bars in the representative comet images are 10 μm. (**D**) Immunofluorescence of 8-oxoguanosine (8-OxoG) from NTP- and NTPO-treated SK-MEL2 cells. Bars and error bars are presented as mean ± SD from three independent experiments (ns = no significant difference; * = *p* < 0.05; ** = *p* < 0.01; *** = *p* < 0.001).

In accord with the notion that ROS are considered the key players in plasma-induced biological effects [4, 7], we previously demonstrated that the addition of oxygen gas flow during NTP treatment accelerated intracellular ROS generation in human lung carcinoma A549 cells, which was dependent on the dose of oxygen gas and in turn induced p53 phosphorylation [9]. However, it has not yet been tested whether the efficiency of NTPO on cancer cell apoptosis is better than NTP alone. Compared to NTP, NTPO produced approximately 3-fold more cleaved-caspase 3 (Figure 1A) and 2-fold more TUNEL positive (Figure 1B) cells in A549 and SK-MEL2 (melanoma) human cancer cells, indicating that the addition of oxygen gas during NTP treatment could increase the apoptotic efficiency, presumably due to accelerated oxyradical production. ROS have been considered important mediators of DNA damage after plasma treatment [9]. Therefore, we decided to investigate the underlying mechanisms of NTPO-induced DDR, which might provide some insights into enhancing NTP efficiency during cancer therapy. First, we asked whether NTP or NTPO could generate substantial genomic DNA lesions and breaks. In both A549 and SK-MEL2 cells, the phosphorylation of a variant histone H2AX (γH2AX), a general marker for DNA breaks, appeared after 2 h in both NTP- and NTPO-exposed cells, whereas cells exposed to a gas (helium) control exhibited no significant γH2AX phosphorylation (Figure 2A). Using a comet assay, we detected the so-called ‘comet-shaped nuclei’ as a result of DNA breaks in the NTP- and NTPO-treated cells under alkaline condition for detection of both DNA single-strand and double-strand breaks (Figure 2B). However, no significant DNA fragmentation was observed under neutral condition for detection of only DNA double strand breaks (Figure 2C), suggesting that NTP and NTPO primarily generate DNA single strand breaks. Further, the quantitative analysis indicated that NTPO produced approximately 2-fold stronger γH2AX phosphorylation and 3-fold more comet nuclei than NTP (Figure 1A and 1B). In accord with the assumption that ROS plays a key role during NTP-induced genotoxicity, we detected 8-OxoG staining from NTP- and NTPO-treated cells in the nucleus as well as the cytoplasm (Figure 2D).

**Figure 3:**
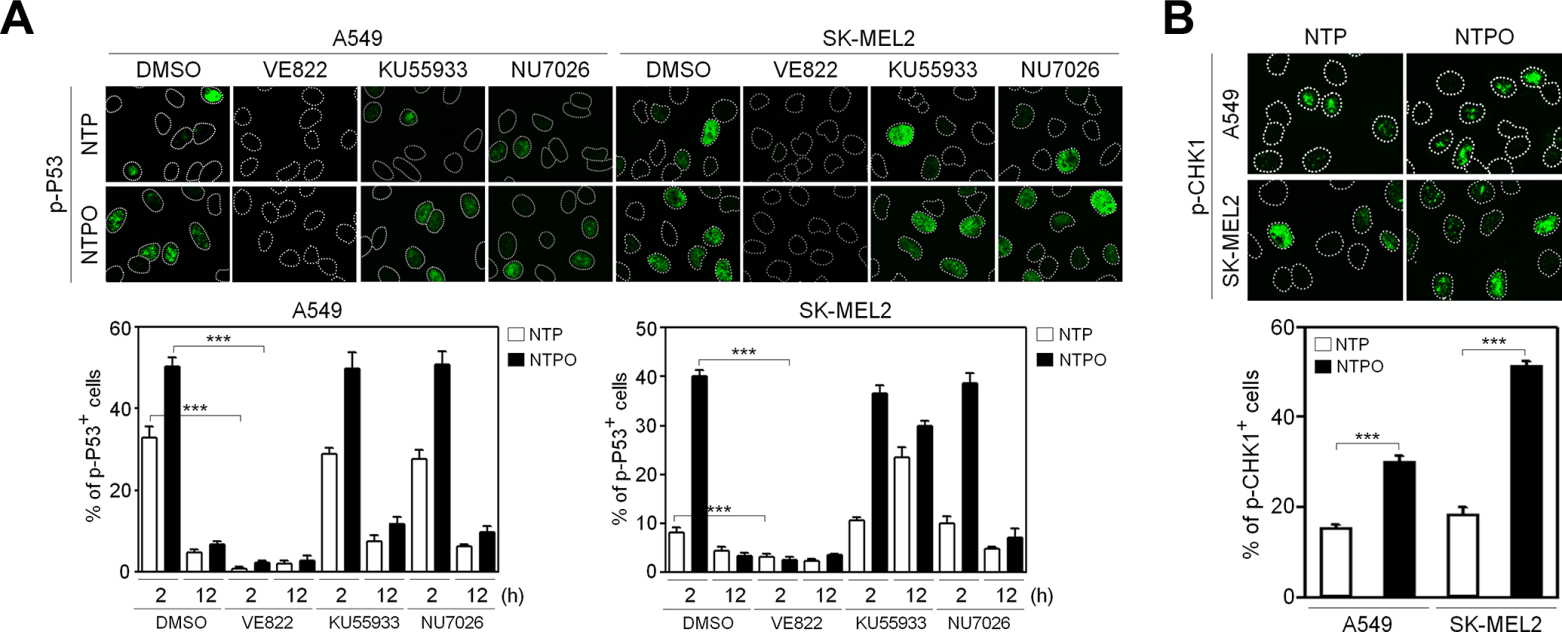
Activation of the ATR-CHK1 pathway in response to NTP-induced DNA damage response (**A**) Phosphorylation of p53 as a marker for NTP- and NTPO-induced DNA damage response was analyzed in the presence of inhibitors targeting ATR (VE822), ATM (KU55933), and DNA-PK (NU7026) before 30 min of plasma treatment in A549 and SK-MEL2 cells. (**B**) Phosphorylation of CHK1 at S345 and S317 was analyzed in response to NTP and NTPO treatment. Bars and error bars represent the mean ± SD from three independent experiments (*** = *p* < 0.001).

**Figure 4:**
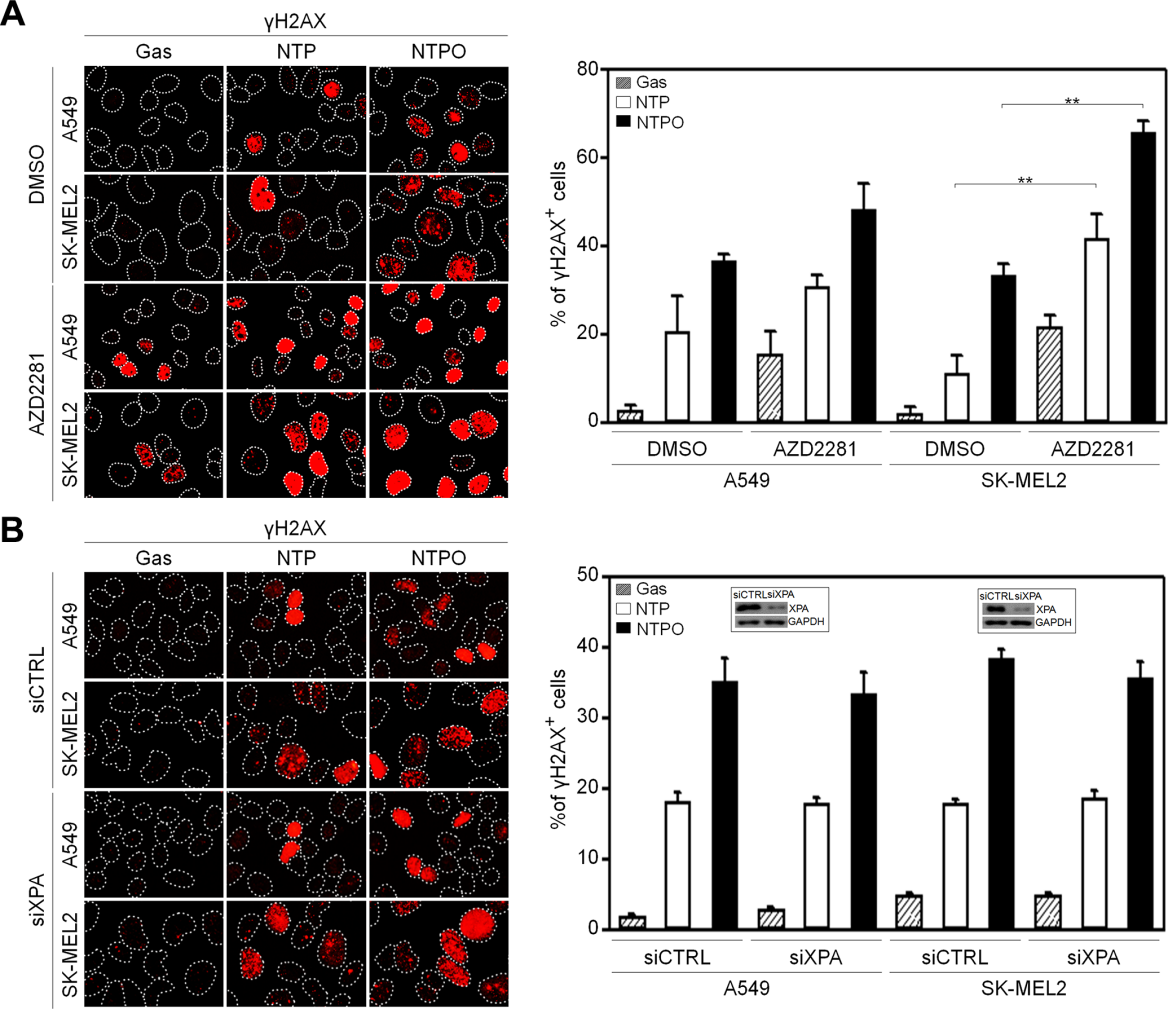
Reinforced DNA breaks in plasma treatment with a PARP inhibitor (**A**) A549 and SK-MEL2 cells pretreated with a PARP inhibitor (AZD2281) or solvent control (DMSO) were stained for γH2AX, and Hoechst-stained nuclei were depicted as dotted lines. (**B**) A549 and SK-MEL2 cells transfected with siXPA or control siRNA (siCTRL) were stained for γH2AX and Hoechst-stained nuclei were depicted as dotted lines. Bars and error bars represent the mean ± SD from three independent experiments (** = *p* < 0.01).

**Figure 5:**
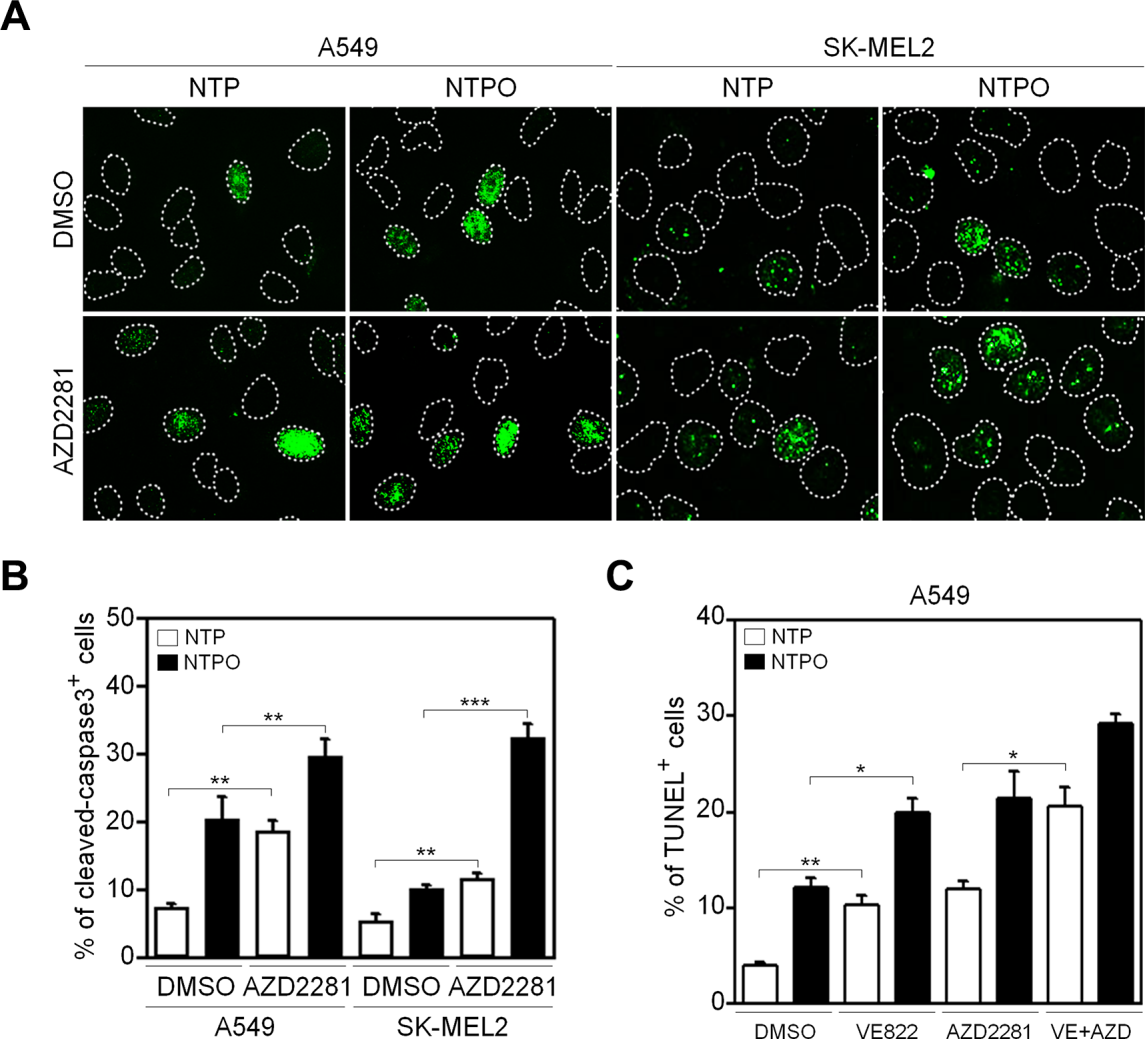
Inhibition of PARP1 augments apoptosis during NTP and NTPO treatment (**A**) A549 and SK-MEL2 cells pretreated with a PARP inhibitor during NTP and NTPO treatment were stained for cleaved-caspase 3, and Hoechst-stained nuclei were depicted as dotted lines. (**B**) The quantitative analysis for (A). The bars and error bars represent the mean ± SD from three independent experiments. (**C**) A TUNEL assay was performed in A549 cells pretreated with ATR, PARP, or ATR + PARP inhibitor during NTP and NTPO treatment. Bars and error bars represent the mean ± SD from three independent experiments (* = *p* < 0.05; ** = *p* < 0.01; *** = *p* < 0.001).

In order to determine the key signaling kinase mediating NTP- or NTPO-induced DDR, the cells were pretreated with specific inhibitors for ATR (VE822), ATM (KU55933), and DNA-PK (NU7026). In mammals, these three kinases represent immediate-early sensors that orchestrate DDR as they commit cell-cycle arrest to secure time for DNA repair in response to genotoxic stresses. As shown in Figure 3A, both NTP- and NTPO-induced p53 phosphorylation was completely abolished in the presence of VE822 in A549 and SK-MEL2 cells. ATR transmits damage signals by phosphorylating CHK1 at Ser317/345, which is essential for cell-cycle arrest in response to genotoxic stresses [17]. Indeed, we could detect CHK1 phosphorylation at both residues upon NTP, which was further potentiated by the addition of oxygen gas flow during NTP treatment (Figure 3B). These results suggested that ATR was the bona fide kinase that mediated the NTP-induced checkpoint activation. Next, we sought to discover the major DNA repair pathway involved in neutralizing NTP-induced DNA damage, which might help enhance NTP efficiency if we could pharmacologically target the pathway during NTP treatment. To this end, we analyzed two DNA repair pathways known to regulate oxidative DNA damage. BER is considered the primary mechanism for removing oxidized bases, which requires the action of PARP1, as indicated by the finding that lysates from PARP1-deficient fibroblasts compromise BER activity when compared with PARP1-proficient cell lysates [15]. As shown in Figure 4A, NTP- and NTPO-induced γH2AX phosphorylation was significantly increased in the presence of AZD2281, a specific inhibitor for PARP1, both in A549 and SK-MEL2 cells. Notably, the phosphorylation of γH2AX, which is normally undetectable in the gas control (DMSO), was also detected in the gas control in the presence of AZD2281 (Figure 4A), which implied the role of PARP1 in the protection of the cancer genome from endogenous DNA damage. However, when we blocked the NER pathway by knocking-down XPA, the key factor for NER mechanisms, no obvious change in γH2AX phosphorylation, compared to the control siRNA transfection, was detected during NTP or NTPO treatment (Figure 4B). Pharmacological inhibition of PARP1 activity increased the apoptotic signal deduced from cleaved-caspase 3 staining (Figure 5A and 5B) and the TUNEL assay (Figure 5C) after 24 h following NTP or NTPO exposure. Further, the apoptotic efficiency of NTPO, which was typically equivalent to the efficiency of NTP in the presence of an ATR or PARP1 inhibitor, was further increased by the addition of an ATR or PARP1 inhibitor (Figure 5C). Importantly, we detected a significant additive effect when ATR and PARP1 inhibitors were co-administered during NTP-induced apoptosis, but the effects were only marginal during NTPO-induced apoptosis (Figure 5C).

**Figure 6:**
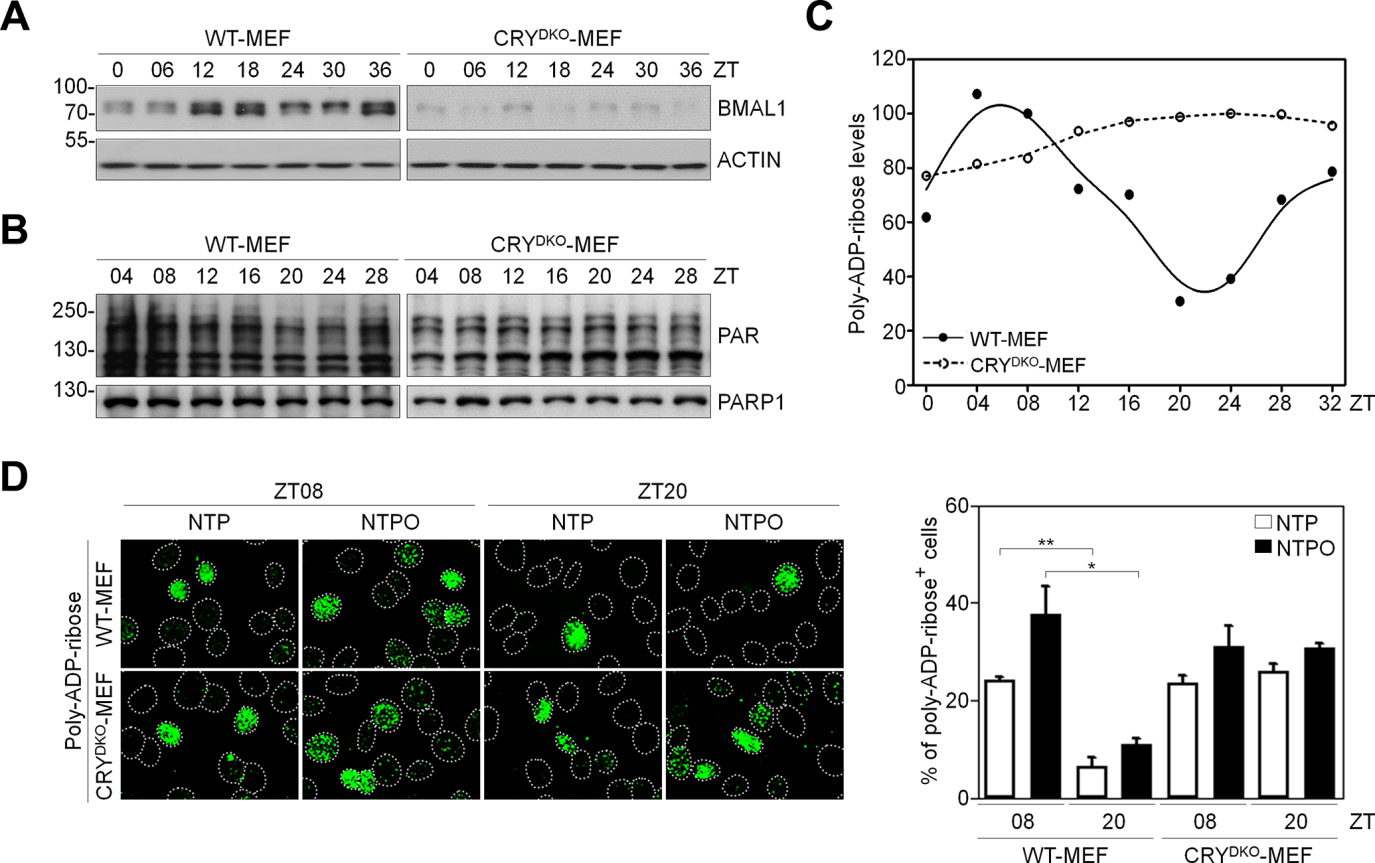
Circadian oscillation of PARP1 activity in normal fibroblasts (**A, B**) Protein levels from forskolin-induced circadian-synchronized WT-MEF and CRY^DKO^-MEF were analyzed by western blotting with the indicated antibodies. (**C**) Quantitative analysis of poly-ADP-ribose (PAR) levels measured in (B). Data points represent averages from three independent experiments. (**D**) Immunofluorescence of poly-ADP-ribose (PAR) after plasma treatment in forskolin-induced circadian-synchronized WT-MEF and CRY^DKO^-MEF. Nuclei were counterstained with Hoechst dye and depicted as dotted lines. Bars and error bars represent the mean ± SD from three independent experiments (* = *p* < 0.05; ** = *p* < 0.01).

**Figure 7:**
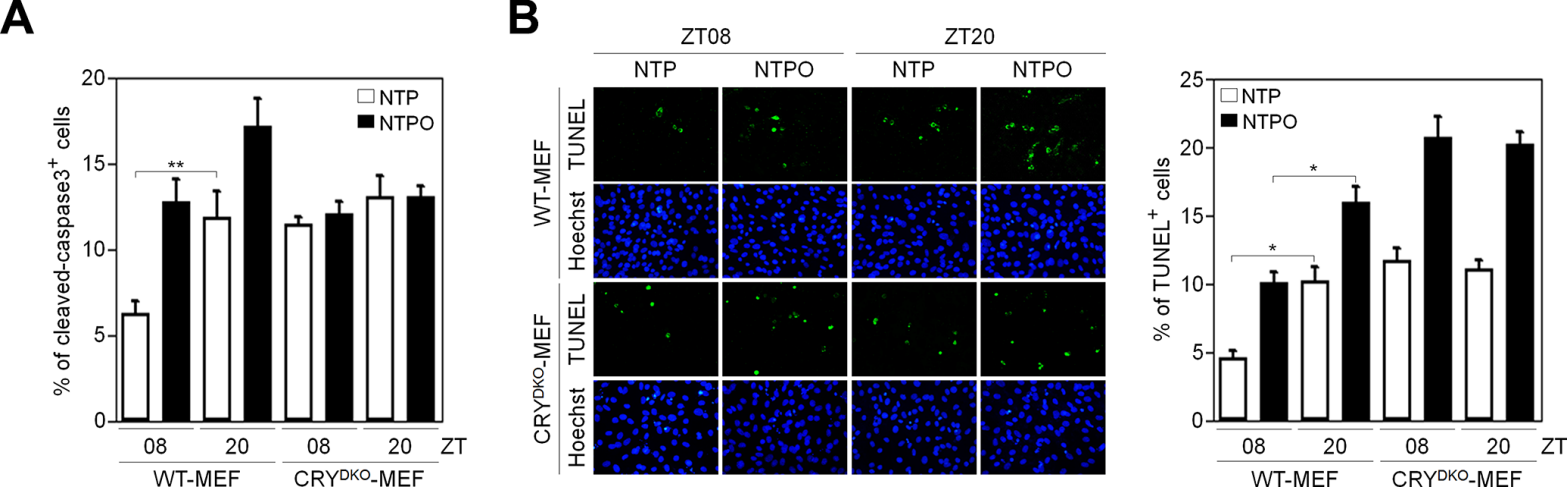
PARP1 activity dictates circadian toxicity of NTP and NTPO in normal cells (**A, B**) WT-MEF and CRY^DKO^-MEF cells exposed to either NTP or NTPO at the indicated ZTs were analyzed for cleaved-caspase 3 (A) and TUNEL signal (B). Bars and error bars represent the mean ± SD from three independent experiments (* = *p* < 0.05; ** = *p* < 0.01).

It has been shown that PARP1 activity has a circadian rhythm in normal mouse cells [18]. In order to investigate the role of PARP1 in NTP-induced genotoxicity in normal cells, which could provide insight into minimizing side-effects to normal tissues during NTP treatment, we employed mouse embryonic fibroblasts that had a functional circadian clock (WT-MEF) and a nonfunctional clock due to the loss of the core clock components cryptochrome 1 and 2 (CRY^DKO^-MEF). To generate a circadian rhythm of gene expression in fibroblasts, we used the forskolin-induced synchronization method as previously described [19]. As shown in Figure 6A, forskolin treatment generated a robust circadian oscillation of a clock-controlled gene BMAL1 as a readout for clock activity in WT-MEF, but not in CRY^DKO^-MEF. Importantly, we found that PARP1 activity deduced from total PAR levels showed a circadian rhythm in WT-MEF but not in CRY^DKO^-MEF, whereas PARP1 protein levels remained unchanged (Figure 6B). To determine whether clock activity affected PARP1 activity during plasma treatment, the cells were treated with NTP or NTPO at ZT08 (ZT0: zeitgeber time 0 at the time of forskolin treatment) and at ZT20 when the PAR signal was maximal and minimal, respectively, during forskolin-induced circadian synchronization (Figure 6C). In WT-MEF, approximately 5-fold more cells exhibited PAR positivity at ZT08 than at ZT20, whereas differential circadian PARP1 activity following NTP or NTPO treatment was not detected in CRY^DKO^-MEF, which exhibited an equivalent number of PAR-positive cells regardless of the ZTs (Figure 6D). Accordingly, cell viability following NTP or NTPO treatment revealed fewer apoptotic cells at ZT08 than at ZT20 in WT-MEF (Figure 7A and 7B), whereas the number of apoptotic cells in CRY^DKO^-MEF was similar between the cells treated with NTP or NTPO at ZT08 and at ZT20. These results indicated that patients' circadian rhythm needs to be considered during NTP or NTPO treatment in order to minimize the side-effects from normal cell damage. Collectively, our results suggested that NTP- or NTPO-induced DNA damage activated ATR-mediated cell-cycle checkpoints and the PARP1-dependent repair pathway, as well as provided an implication for chronoplasma therapy.

## DISCUSSION

### NTP-induced DDR

To improve the therapeutic efficacy of NTP during cancer treatment, we investigated NTP and NTPO-induced DDR, including cell-cycle checkpoints, DNA repair, and apoptosis. Our results indicated that NTP or NTPO treatment in human cancer cells could induce ATR-mediated cell-cycle checkpoints and PARP1-dependent DNA repair. Therefore, the apoptotic efficiency of NTP could be enhanced when it is combined with either ATR or a PARP1 inhibitor. It has been shown that targeting the ATR pathway is a promising strategy for cancer therapy [20]. In fact, ATR inhibitors are undergoing clinical trials in combination with DNA-damaging chemotherapy and ionizing radiation [21, 22]. PARP inhibition, which impairs DNA repair activity, has been exploited as an anticancer strategy to increase the cytotoxicity of chemotherapeutics and radiotherapy [23–25]. Several clinical trials are in progress for the treatment of breast and ovarian cancers, and the PARP inhibitor olaparib (AZD2281) has recently been approved for the treatment of advanced ovarian cancers [26]. As well, NTP can easily be applied to superficial cancers such as skin and breast cancer. Endoscopic NTP is currently under development as a means to reduce the barriers to NTP target cancers and could eventually be applied in the treatment of almost every cancer type, including cancers that originate internally [1]. Compared to radiation therapy, which primarily derives its efficacy by efficiently generating double-strand DNA breaks as well as high mutational rates, NTP produces single-strand DNA breaks with moderate efficiency and lower mutational rates. Therefore, based on our findings, a combinatorial regimen targeting ATR and PARP1 pathways during NTP treatment might have the potential for development as an enhanced cancer therapy with favorable therapeutic responses.

### Chronoplasma therapy

The circadian timing system is composed of molecular clocks [27] that drive 24 h changes in almost all cellular physiologies including the cell cycle, DNA repair, and apoptosis [28–31]. It has been shown that circadian timing can modify the tolerability of anticancer medications 2- to 10-fold in experimental models, as well as in cancer patients [32]. Chronotherapeutics aims to improve the tolerability and/or the efficacy of medications through the administration of treatments according to biological rhythms. The adequate adjustment of treatment delivery to account for physiological rhythms and the restoration or the induction of these rhythms can improve therapeutic outcomes in cancer patients. In this report, we found that PARP1 activity in normal fibroblasts had a circadian rhythm, which was consistent with previous data showing circadian PARP1 activity in the mouse liver [18]. As well, the circadian activity of PARP1 significantly affected cell viability following NTP or NTPO-induced DNA damage responses. Thus, our findings could be beneficial for designing mechanism-based chronoplasma trials.

## MATERIALS AND METHODS

### Plasma treatment

The plasma jet device reported previously [9] was used to treat cells. A typical operating condition of the pulsed-dc plasma jet has the applied voltage 1.6 kV, repetition frequency 50 kHz, and duty ratio 10%. The working gas (helium) and reactive gas (oxygen) flow rate were kept constant at 500 and 5 SCCM (SCCM denotes cubic centimeter per minute at standard temperature and pressure), respectively. To produce more radicals in the gas phase, our plasma sources was consist of injected oxygen gas into the capillary tube while helium was fed through the quartz tube because adding O_2_ directly into the feeding gas tends to diminish the production of radicals due to the electron attachment to oxygen inside the nozzle. The cells were cultured on 12-mm microscope cover glass coated with gelatin from porcine skin (Sigma-Aldrich) and were treated with helium generated-NTP with or without the flow of oxygen gas. The exposed cover glass was then transferred to 12-well plates containing fresh medium and incubated at 37°C in a humidified incubator supplemented with 5% CO_2._ If necessary, the cells were treated with 5 or 10 μM of specific inhibitors targeting ATR (VE822), ATM (KU55933), DNA-PK (NU7026), or PARP1 (AZD2281) for 30 min before plasma treatment and kept until cell harvest. The inhibitors were purchased from Selleck Chemicals.

### Cell culture, siRNA transfection, and circadian synchronization

Wild-type mouse embryonic fibroblasts (WT-MEF) and CRY1 and CRY2 double knockout mouse embryonic fibroblasts (CRY^DKO^ MEF, a gift from Dr. KJ Kim, Seoul National University) were cultured in Dulbecco's Modified Eagle's Medium (Hyclone) supplemented with 10% fetal bovine serum (Hyclone) and 1% penicillin-streptomycin (Hyclone). Human lung carcinoma A549 and melanoma SK-MEL2 cells were cultured in Dulbecco's Modified Eagle's Medium supplemented with 10% fetal bovine serum and 1% penicillin-streptomycin and in Eagle's Minimum Essential Medium (Hyclone) supplemented with 10% fetal bovine serum, 1% penicillin-streptomycin, 1% sodium pyruvate (Gibco), and 1% non-essential amino acids (Gibco), respectively. If necessary, the cells were transfected with siRNA duplexes targeting XPA (Dharmacon) using Lipofectamine^®^ 2000 (Invitrogen) according to the manufacturer's protocol. After 48 h of siRNA transfection, 1.6 kV of NTP or NTPO was treated onto A549 and SK-MEL2 cells and followed by an analysis for immunostaining of γH2AX. For circadian synchronizations, MEF cells were treated and cultured as previously reported [19].

### Immunofluorescence

For immunofluorescence staining, cells were fixed with 4% paraformaldehyde (Sigma-Aldrich) for 10 min at room temperature and permeabilized with 0.5% Triton^™^ X-100 (Bio Basic). Specific antibodies against 8-OxoG (Abcam), phospho-histone H2AX (Ser139; Millipore), phospho-CHK1 (Ser345), phospho-P53 (Ser15), PARP1, cleaved-caspase 3 (Cell Signaling Technology), and poly-ADP-ribose (Enzo Life Sciences) were used for visualization of the proteins. The images were captured using a fluorescence microscope (Nikon) equipped with the NIS-Elements 4.0 Nikon imaging software. For quantification, a minimum of 500 cells were analyzed from each of three independent experiments.

### Immunoblotting

Immunoblotting was performed as described previously [33]. Briefly, collected cells were resuspended in 100 μl of 1X lysis buffer (20 mM Tris-HCl pH 6.8, 150 mM NaCl, 1 mM EDTA, 1 mM EGTA, protease inhibitor cocktail, and 10% Triton^™^ X-100) and sonicated using a sonicator (SONICS). Total protein (30 μg) was run on 10% SDS-polyacrylamide gels and transferred to nitrocellulose blotting membranes using electrophoresis chambers (Bio-RAD Laboratories). Membranes were analyzed by immunoblotting with antibodies for XPA (Kamiya Biomedical Company), CRY1, BMAL1 (Santa Cruz Biotech), and GAPDH (Cell Signaling Technology).

### Comet assay

DNA breaks were determined using the CometAssay^®^ Kit (Trevigen). Briefly, after 24 h of plasma treatment, the cells were mixed at a 1: 10 (v/v) ratio with low-melting-point agarose at 37°C. The cell suspension (75 μl) was dispersed onto a microscope comet slide and maintained at 4°C for 40 min. The cells were then lysed at 4°C and incubated either in alkaline solution (200 mM NaOH and 1 mM EDTA) to detect both DNA single strand and double strand breaks, or incubated in neutral electrophoresis buffer (100 mM Tris-HCl, 300 mM sodium acetate) to detect double stand breaks. The slides were then placed and run in a horizontal electrophoresis system. Afterward, the slides were gently immersed twice in dH_2_O for 5 min each and in 70% ethanol for 5 min, and then dried overnight at room temperature. Before comet scoring, the DNA was counterstained with SYBR^®^ green for visualization under a fluorescence microscope. The tail moment was calculated using Comet Assay Score software Project (CASP) software.

### Terminal deoxynucleotidyl transferase dUTP nick end-labeling (TUNEL) assay

For the detection of DNA fragmentation, the Click-iT^®^ TUNEL Alexa Fluor^®^ Imaging Assay (Invitrogen) was used according to the manufacturer's instructions. Briefly, cells were fixed in 4% paraformaldehyde for 10 min at room temperature and permeabilized in 0.5% Triton^™^ X-100 in PBS for 20 min at room temperature. The cells were then incubated for 60 min at 37°C in terminal deoxynucleotidyl transferase enzyme reaction mixture. The cells were washed twice with 1X PBST for 2 min each and then incubated for 30 min at room temperature with Click-iT^®^ reaction mixture. Cell nuclei were counterstained with Hoechst 33342 (Sigma-Aldrich) for 15 min at room temperature and images were taken under a fluorescence microscope.

### Statistics

Statistical significance was determined using Student's *t*-test. Bars and error bars were presented as mean ± SD from at least three independent experiments. Differences were considered significant at the values of *p* < 0.05 (*), *p* < 0.01 (**), and *p* < 0.001 (***). All statistical analyses were performed using the GraphPad Prism 5.0 software (GraphPad).
